# Comparative phenotyping of C57BL/6J substrains reveals distinctive patterns of cardiac aging

**DOI:** 10.1007/s11357-025-01543-7

**Published:** 2025-01-30

**Authors:** Sophia Walter, Patricia Baumgarten, Niklas Hegemann, Steffen P. Häseli, Stefanie Deubel, Julia Jelleschitz, Annika Höhn, Nikolaus Berndt, Wolfgang M. Kuebler, Jana Grune, Christiane Ott

**Affiliations:** 1 Molecular Toxicology, German Institute of Human Nutrition Potsdam-Rehbruecke (DIfE), Nuthetal, Germany; 2 TraceAge-DFG Research Unit On Interactions of Essential Trace Elements in Healthy and Diseased Elderly, Potsdam, Germany; 3 DZHK (German Center for Cardiovascular Research), Partner Site Berlin, Berlin, Germany; 4 Institute of Physiology, Charité – Universitätsmedizin Berlin, corporate member of Freie Universität Berlin and Humboldt-Universität Zu Berlin, Charitéplatz 1, 10117 Berlin, Germany; 5 Department of Cardiothoracic and Vascular Surgery, Deutsches Herzzentrum Der Charité (DHZC), Augustenburger Platz 1, 13353 Berlin, Germany; 6 Charité-Universitätsmedizin Berlin, corporate member of Freie Universität Berlin and Humboldt-Universität Zu Berlin, Charitéplatz 1, 10117 Berlin, Germany; 7 German Center for Diabetes Research (DZD), Munich, Germany; 8 Institute of Computer-Assisted Cardiovascular Medicine, Deutsches Herzzentrum Der Charité (DHZC), Augustenburger Platz 1, 13353 Berlin, Germany

**Keywords:** Heart failure, Cardiomyocyte, Echocardiography, Proteomics, Cardiac aging phenotype

## Abstract

Research in aging often refers to animal models, particularly C57BL/6J (B6J) mice, considered gold standard. However, B6J mice are distributed by different suppliers, which results in divers substrains exhibiting notable phenotypic differences. To ensure a suitable phenotype of cardiac aging, we performed heart analyses of young (5 months) and old B6J mice (24 months) from two substrains: B6JRj (Janvier) and B6JCrl mice (Charles River). In hearts of both substrains, myocardial fibrosis increased with age; however, only in old B6JRj mice cardiac hypertrophy associated with a decreased ejection fraction was observed. Gene set enrichment analysis in heart tissue using proteomic data revealed different age-associated pathway changes between the substrains, especially in oxidative phosphorylation. Functional assessment of isolated cardiomyocytes verified cardiac impairment during aging in B6JRj mice. Overall, results demonstrate that cardiac aging manifests as a moderate systolic dysfunction in B6JRj mice, while B6JCrl mice display no functional changes with age.

**Supplementary Information:**

The online version contains supplementary material available at 10.1007/s11357-025-01543-7.

## Introduction

Cardiovascular diseases (CVDs) are the leading cause of death globally. During aging, the risk for CVDs increases dramatically. Accordingly, 89.4% of males and 90.8% of females aged 80 years or older suffer from CVDs [1]. Structural changes such as an increase in left ventricular (LV) mass and LV thickness are associated with physiological aging. However, also pathological changes of the heart are frequently observed phenotypes in the elderly, including LV hypertrophy, fibrosis, and diastolic dysfunction [2]. Such pathological changes are often related to heart failure (HF), a clinical syndrome that increases in prevalence with age and is associated with CVDs [3]. HF is defined as structural or functional impairment of blood filling or ejection of the heart leading to symptoms or signs such as fatigue or dyspnoea during physical activity. To confirm HF in patients with associated symptoms, diagnosis is often based on ejection fraction (EF) in context with elevated natriuretic peptides, e.g. n-terminal pro B-type natriuretic peptide (NT-proBNP), or congestion [4]. In addition to natriuretic peptides, neurohormonal markers can be used as an indicator for HF, since a reduced heart function is also associated with elevated levels of, e.g. norepinephrine [5]. At molecular level, cardiac aging proceeds slowly and is driven by oxidative stress and dysfunctional mitochondria [6], leading to an impaired energy metabolism of cardiomyocytes. This impairment shows up as the loss of cardiac metabolic flexibility, resulting in enhanced glucose and decreased fatty acid utilization [7] as well as the decreased activity of the oxidative phosphorylation (OXPHOS) pathway causing an energy deficit, impairing cardiac function and contributing to CVD [7].

Studies on cardiac aging and associated phenotypes critically rely on suitable and well-established animal models which mimic the clinical phenotype of interest as closely as possible. The inbred C57BL/6 (B6) mouse strain has become the gold standard for most animal experiments. It originated from C57BL mice, established by Dr. C. C. Little in 1921 [8]. Since then, various substrains were created by breeding the mice at different laboratories. As a result, at least 3000 B6 substrains have been identified up to now. These substrains differ in fundamental characteristics such as behaviour, metabolism, but also in the cardiovascular system [8]. Reasons for these differences are often attributed to single nucleotide polymorphisms [9, 10], but variations in housing conditions may also impact on phenotypes [11]. In 1948, mice B6 were established at Jackson Laboratory (B6J) and further distributed to the National Institute of Health in 1951 (B6N) [8]. While the B6N mice contain the gene encoding the nicotinamide nucleotide transhydrogenase, most B6J mice show a functional deletion for this gene [10], resulting in an altered glucose tolerance and impaired mitochondrial function when compared to B6N mice [12]. While the differences between B6J and B6N mice are well understood, studies concentrating on the differences between B6J substrains from different vendors are largely lacking. In one of the few studies focusing on such substrain characteristics, Siersbæk et al. (2020) showed that B6J substrains from three different vendors responded differently to high-fat diet. Specifically, B6JCrl mice from Charles Rivers and B6JRj mice from Janvier gained a higher percentage of fat tissue compared to B6JBomTac mice from Taconic Bioscience, with B6JCrl mice showing the strongest increase [13].

In the past, studies on heart function in aging mice have led to inconsistent results. While in some studies cardiac aging was associated with declining heart function [14, 15], others failed to detect cardiac impairment with age in mice [16, 17]. We speculated that some of this heterogeneity is attributable to substrain differences, and specifically hypothesized that discrepancies at metabolic level between B6JCrl and B6JRj mice affect cardiac function during aging. To test this hypothesis, we analyzed whether B6J mice age differently based on their substrain. We focused on B6JCrl mice from Charles Rivers and B6JRj mice from Janvier. While Charles River is the European vendor of Jackson mice (B6JCrl), Janvier distributes B6JRj mice since 1993 [10]. We chose these two substrains, since both are frequently used for genetic models and metabolic studies [8, 18] and are already known to express notable differences in their metabolic phenotype [13]. We characterized the cardiac aging phenotype of the substrains by investigating structural, functional, and proteome alterations in hearts of young and old mice of both substrains. Using this approach, we were able to characterize a reliable model for the study of the cardiac aging phenotype with systolic impairment in vivo. Analyses of the cellular and molecular mechanisms underlying substrain differences in cardiac aging may provide a powerful tool to identify mechanisms of cardiac aging and age resistance with potential therapeutic implications.

## Methods

### Animal models

Male B6JRj mice were originally obtained from Janvier Lab. Male B6JCrl mice were originally obtained from Charles River. Mice from both substrains were bred in-house. At least every 20 generation mice were back crossed to prevent the development of a new substrain. To rule out significant breeding differences, B6JRj mice for echocardiography analysis were directly obtained from Janvier. Young mice were sacrificed with 5 months of age, old mice with 24 months of age. Individual mice were sacrificed for the experiments (see figure legend for corresponding sample size). Mice were housed at 40–50% humidity and 22 ± 2 °C under 12-h light/dark cycle with water and food ad libitum. All mice received a standard rodent chow (V1534, Ssniff Spezialdiäten GmbH). All animal procedures were performed in accordance with the “Guide for the Care and Use of Laboratory Animals” (Institute of Laboratory Animal Resources, 7th edition 1996) and the European legislation for animal welfare (Directive 2010/63/EU). All procedures were approved by the local authorities (LAGESO, G0239/16 and LAVG, 2347–46–2019) and in accordance with institutional guidelines. Information regarding genetic background of both substrains is available in the supplementary material (GVG Genetic Monitoring GmbH, Leipzig, Supplements Table 1-3)

### Echocardiography

Echocardiography was performed as described previously using a Vevo 3100 high-resolution imaging system coupled to an MX400 ultra-high-frequency linear array transducer (18–38 MHz; centre transmit: 30 MHz; axial resolution: 50 µm; both FUJIFILM VisualSonics) [19]. Mice were exposed to 3% isoflurane (Baxter International, Deerfield, IL) followed by fixation on a heating pad in dorsal position. Images were taken in parasternal long axis view. M-mode was used for analysis of morphological LV changes, and B-mode was used for functional assessment of the heart. Images were stored in raw format (DICOM). A trained expert conducted additional offline analyses by applying the software package VevoLAB Version 3.1.0 (FUJIFILM VisualSonics). After the experiment, animals were sacrificed by cervical dislocation and the hearts were collected.

### Histological stainings

For immunohistochemical staining, cross-sectional 2-µm-thick paraffin slices were processed. Sections were fixed in 4% formalin (Thermo Scientific) followed by storing in 70% ethanol. For imaging, Axio Scan 7 (Zeiss) with 20 × objective was used for fine focus, and 5 × objective was used for coarse. Analysis was performed using Zeiss ZEN 3.0 (blue edition) and Zeiss ZEN Imaging Software.

Sections were stained with picrosirius red. Total collagen levels were calculated by dividing the red-stained collagen area by total tissue area. Mean vascular collagen area was evaluated by quantifying the red stained around 10 vessels per heart cross section with similar size and location.

CD45 was stained via immunofluorescence. Paraffin slices were deparaffinized followed by an antigen retrieval using citric buffer [10 mM citric acid, 0.05% Tween20®; pH = 6.0]. Blocking was performed with 10% goat serum in antibody diluent (S080983-2, Agilent Technologies). Antibody CD45 (559,864, BD Bioscience) was diluted 1:50 in blocking buffer and incubated overnight at 4 °C. After washing, slices were incubated for 1 h with secondary antibody Alexa Fluor 633 (A-21094, Invitrogen) and DAPI (5 µg/ml, D9542, Sigma). Slices were washed and mounted with Fluoromount-G™ Mounting Medium (00–4958-02, Invitrogen). For analysis, the number of DAPI-positive nuclei was counted, and for each nucleus, the mean and maximum intensity of CD45 was determined. We used a cutoff for both intensities to calculate the percentage of CD45-positive cells. For the cutoff, we took the respective background staining of CD45 and the histogram of CD45 intensity in DAPI areas into account.

### Plasma marker

Measurement of plasma norepinephrine, plasma NT-proBNP, and plasma insulin was performed using enzyme-linked immunosorbent assays (ELISAs) (norepinephrine: KA1877, Abnova; NT-proBNP: NBP2-76,775, Novus; insulin: 80-INSMSU-E01, ALPCO). Analyses of non-esterified fatty acids (NEFAs) and total triglycerides was done by using the Mira Autoanalyzer (Roche) and respective kits (glucose: A11A01667; triglycerides: A11A01640, Axon Lab; NEFAs: 434–91,795, 434–91,995, Wako).

Therefore, blood was taken from vena cava and centrifugated for 5 min at 4 °C at 14,000 rpm. The supernatant (plasma) was collected, snap-frozen in liquid nitrogen, and stored at − 80 °C. For the ELISAs, protocols were performed according to the manufacturer’s instruction.

### Western blot

The apexes were separated from the collected hearts, snap-frozen in liquid nitrogen, and stored at − 80 °C. The heart tissue was lysed in SDS-buffer [10 mM Tris (pH = 7.5), 0.9% NP-40, 0.1% SDS, 1 mM Pefabloc®] containing protease inhibitor (11,873,580,001, Roche) using a ball mill. After centrifugation, the supernatant was collected and protein concentration was determined by Lowry assay. Laemmli buffer [0.25 mM Tris (pH = 6.8), 40% glycerol, 20% 2-mercaptoethanol, 8% SDS, 0.03% Bromophenol Blue] was added to the samples. Twenty micrograms of protein was separated by SDS polyacrylamide gel electrophoresis and transferred to a 0.45-µm nitrocellulose membrane (10,600,002, Cytiva). Ponceau S staining (0.2% Ponceau S, 1% acetic acid) was performed and signal was quantified by a densitometer (GS800, BioRad) using the Quantity One software. Membranes were destained and blocked for 1 h at RT with PBS containing 2% BSA (8076.3, Roth) and 0.01% sodium azide. OXPHOS (ab110413, Abcam) was used as primary antibody diluted 1:250 in blocking buffer with 0.1% Tween® 20 (P9416, Sigma) and incubated overnight at 4 °C. After washing membranes with PBS containing 0.1% Tween® 20, membranes were incubated with a fluorescent-labelled secondary antibody (926–32,210, LI-COR) for 1 h at RT followed by another washing step. Membranes were scanned using the Odyssey® CLx Imaging System (LI-COR) and analyzed by the Image Studio Software (v 5.2, LI-COR). OXPHOS signal was normalized to total membrane protein measured by Ponceau S staining.

### Cardiomyocyte contraction analysis

Cardiomyocytes were isolated based on a Langendorff-free method [20]; the adjusted protocol was described previously [21]. In brief, after anesthetization of the mice using isoflurane, cervical dislocation was performed to sacrifice the mice. The heart was removed and rinsed with EDTA and perfusion buffer, followed by enzymatical and mechanical digestion. Stop solution was applied. Suspension was filtered using 100-µm pore-size strainers. Three rounds of gravity settings and calcium reintroduction were performed. Medium supernatant was discarded and replaced by prewarmed Krebs–Ringer solution (137 mM NaCl, 5.4 mM KCl, 0.5 mM MgSO_4_*7H_2_O, 10 mM D-glucose, 1 mM CaCl_2_*2H_2_O, 0.4 mM K_2_HPO_4_*3H_2_O, 25 mM NaHCO_3_; pH = 7.4). Cells were carefully resuspended, and cell suspension was pipetted in the chamber of the IonOptix system. Cells were settled for 1 min. The prewarmed Krebs–Ringer solution was used in a pumping system to ensure that the cells are exposed to the optimal temperature. After 3 min in the flow, contraction was measured using the IonOptix Myocyte Calcium and Contractility System. Cells were paced with 10 V at 1 Hz. Per mice, at least 10 cells were analyzed; per cell, at least 10 transients were measured. Demonstrated results show the changes in sarcomere length per time. Data was analyzed using the IonOptix program CytoSolver.

### Proteomics

LC–MS/MS was carried out by nanoflow reverse-phase liquid chromatography (Dionex UltiMate 3000, Thermo Scientific, Waltham, MA) coupled online to a Q Exactive HF Orbitrap mass spectrometer (Thermo Scientific, Waltham, MA). The LC separation was performed using a PicoFrit analytical column (75 µm ID × 55 cm long, 15 µm Tip ID (New Objectives, Woburn, MA) in-house packed with 3-µm C18 resin (Reprosil-AQ Pur, Dr. Maisch, Ammerbuch-Entringen, Germany)) as reported previously [22]. Briefly, peptides were eluted using a gradient from 3.8 to 50% solvent B in solvent A over 121 min at 266 nL per minute flow rate. Solvent A was 0.1% formic acid and solvent B was 79.9% acetonitrile, 20% water, and 0.1% formic acid. Nanoelectrospray was generated by applying 3.5 kV. A cycle of one full Fourier transformation scan mass spectrum (300 − 1750 m/z, resolution of 60,000 at m/z 200, AGC target 1e6) was followed by 12 data-dependent MS/MS scans (resolution of 30,000, AGC target 5e5) with a normalized collision energy of 25 eV. To avoid repeated sequencing of the same peptides, a dynamic exclusion window of 30 s was used. In addition, only the peptide charge states between two to eight were sequenced.

Raw MS data were processed with MaxQuant software (1.5.7.4) 6 with the Andromeda search engine 7 and the mouse UniProtKB with 51,416 entries released in 03/2016. A false discovery rate (FDR) of 0.01 for proteins and peptides, a minimum peptide length of seven amino acids, a mass tolerance of 4.5 ppm for precursor, and 20 ppm for fragment ions were required. A maximum of two missed cleavages was allowed for the tryptic digest. Cysteine carbamidomethylation was set as fixed modification, while N-terminal acetylation and methionine oxidation were set as variable modifications.

#### Gene set enrichment analysis (GSEA)

GSEA was performed using the MSigDB and the provided mouse-ortholog hallmark gene sets [23–25]. In this analysis, the hearts of young B6JRj were compared to the hearts of young B6JCrl mice. The FDR was estimated by using gene set-based permutation test (1000 permutations). Only cardiac relevant pathways were embedded. Due to the small sample size (*n* = 4), we only included pathways with FDR/*q*-value ≤ 0.25. Pathways with FDR/*q*-values ≤ 0.05 were stated as significant enriched.

#### CARDIOKIN1

For the quantification of the metabolic changes caused by the abundance changes of metabolic enzymes, we used the kinetic model CARDIOKIN1 [26] which comprises all pathways involved in the catabolism of the energy-delivering substrates glucose, lactate, fatty acids, ketone bodies, and branched chain amino acids. The model takes into account the regulation of metabolic enzymes and transporters by substrate affinities, allosteric regulations, and the short-term regulation by the hormones insulin and catecholamines. The time-dependent variations of model variables (= concentration of metabolites and ions) are governed by first-order differential equations. Time variations of small ions were modelled by kinetic equations of the Goldman-Hodgkin-Katz type [27]. Numerical values for kinetic parameters of the enzymatic rate laws were taken from reported kinetic studies of the isolated enzyme. Maximal enzyme activities (Vmax values) were estimated based on functional characteristics and metabolite concentrations of a healthy tissue [26]. Hormone-dependent regulation of the central energy metabolism by reversible enzyme phosphorylation was taken into account by a phenomenological model of insulin- and epinephrine-dependent changes of plasma glucose and changes in the phosphorylation state of interconvertible enzymes as previously described [26, 28]. Individual model instantiations were generated based on proteomic profiles as described in Berndt et al. [29]. Maximal metabolic capacities were defined by the magnitude of fluxes in response to changes in the concentration of a distinct plasma metabolite, while keeping all other plasma metabolites at a constant level. For a detailed description, see Berndt et al. [26].

### Statistics

Statistical analyses were done using GraphPad Prism (v. 9.5.0). Outliers were removed as indicated by the Rout Outlier test (Q = 1%). Normality was tested using Shapiro Wilk test. Unless otherwise described, unpaired *t*-test (normality passed) or Mann–Whitney *U* test (normality not passed) was performed to compare the young and old group within each substrain. Results are presented as mean ± SD and statistical significance was considered at *p* ≤ 0.05.

## Results

### Age-related changes in heart structure

**Fig. 1:**
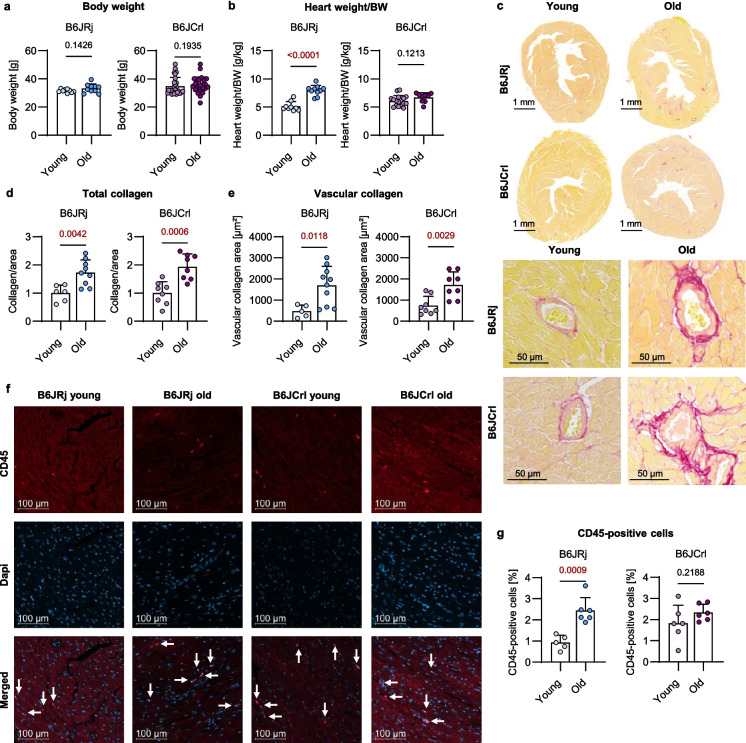
Cardiac aging is associated with structural cardiac changes in two B6J substrains. **a**, **b** Body weight (BW) and heart weight per BW were measured in young (5 months) and old (24 months) mice (*n* = 9–27). **c** Collagen was stained by picrosirius red and representative pictures of the total heart cross section and cardiac vessels are shown. **d**, **e** Collagen area was divided by total area to quantify total collagen and perivascular collagen area was quantified (*n* = 6–10). **f**, **g** CD45 was stained in heart tissue samples. The number of CD45-positive cells was calculated by dividing the CD45-positive nuclei by the total amount of nuclei indicated by DAPI (*n* = 5–6). Data represent mean ± SD. Statistical significance was tested with Student’s *t*-test (normality passed) or Mann–Whitney *U* test (normality not passed) and *p*-values are shown. B6, C57BL/6

To assess the cardiac aging phenotype of B6JRj (Janvier) and B6JCrl (Charles River) mice, males of both substrains were bred in-house to an age of 5 months (young) and 24 months (old). Additional mice were directly purchased from Janvier to exclude in-house breeding effects. We aimed to investigate whether substrain-specific age-associated changes in the hearts of each substrain exist; accordingly, we compared young and old mice within each substrain. The body weight (BW) of the mice was not affected during aging in both substrains (Fig. 1a). The heart weight per BW increased during aging in B6JRj mice but not in B6JCrl mice (Fig. 1b). Collagen levels were analyzed using picrosirius red staining as an indicator for extracellular matrix deposition. The total collagen area was elevated in the hearts of old mice from both substrains compared to young groups (Fig. 1c–d). Additionally, collagen deposition around the vessels was analyzed to quantify vascular fibrosis. In accordance with total collagen levels, the hearts of both substrains showed an increase in vascular collagen with age (Fig. 1c, e). Since collagen deposition is associated with the infiltration of immune cells, we analyzed the number of CD45-positive cells in heart tissue samples. Interestingly, in B6JRj mice, the number of CD45-positive cells increased with age. However, we did not observe the same aging effect in the hearts of B6JCrl mice (Fig. 1f, g).

### Heart function

**Fig. 2:**
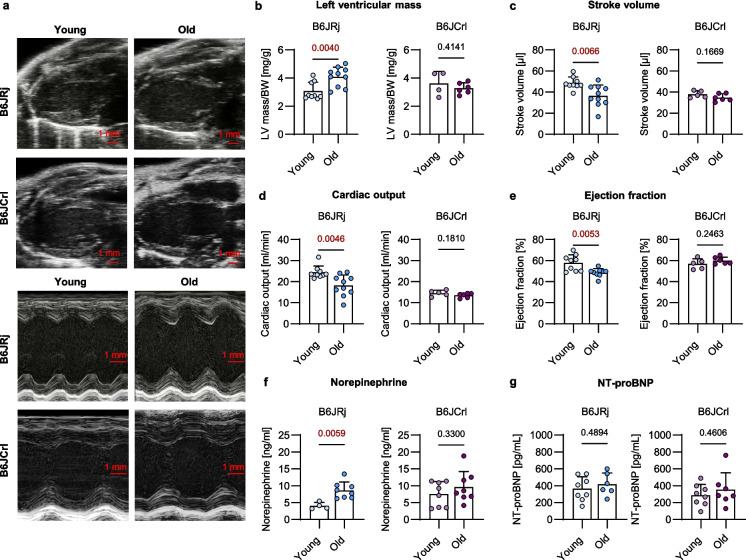
Age-associated decline in heart function is more pronounced in B6JRj than in B6JCrl mice. **a**, **b**, **c**, **d**, **e** Left ventricular (LV) mass per body weight (BW), stroke volume, cardiac output, and ejection fraction were analyzed by echocardiography in young (5 months) and old (24 months) anesthetized mice (n = 4–10). **f**, **g** Plasma norepinephrine and n-terminal pro b-type natriuretic peptide (NT-proBNP) were determined using commercial-available ELISAs (*n* = 4–8). Statistical significance was tested with unpaired *t*-test (normality passed) or Mann–Whitney *U* test (normality not passed) and *p*-values are shown. B6, C57BL/6

Echocardiography was performed to further estimate how and to what extent aging alters the functional cardiac phenotype in the two B6J substrains. Contrary to the total group of mice, B6JCrl mice used in echocardiography analyses showed an increase in BW with age (Table 1). Age-associated changes in LV size were only present in B6JRj mice (Fig. 2a), in those LV mass per BW increased with age (Fig. 2b). LV anterior wall (LVAW) and LV posterior wall (LVPW) thickness during both systole and diastole increased with age in B6JRj mice, while the systolic and diastolic LV diameters were not altered. In B6JCrl mice, LV thicknesses did not change during aging. Additionally, end-diastolic volume (EDV) and end-systolic volume (ESV) did not differ between the age groups of B6JRj mice, while B6JCrl mice showed a significant reduction in EDV and a similar trend for ESV during aging (*p* = 0.07; Table 1). In B6JRj but not in B6JCrl mice, stroke volume and cardiac output declined with age (Fig. 2c–d). Since stroke volume and cardiac output increase in obese individuals, the age-associated increase in BW in B6JCrl mice might impact the results in the B6JCrl group [30]. Therefore, we correlated functional readouts with BW in old B6JCrl mice. Yet, no significant correlation between stroke volume (R^2^= 0.5006, *p* = 0.1158) or cardiac output with BW (R^2^ = 0.4060, *p* = 0.1736, Supplements Fig. 1) was observed. In accordance with the decrease in cardiac output and stroke volume in the B6JRj group, the EF decreased with age in these mice, reaching a value of 48.6%, while EF was not altered in B6JCrl mice (Fig. 2e). Since the reduction in EF suggests the development of HF in the old B6JRj group, we measured norepinephrine and NT-proBNP as plasma markers for HF [5]. During aging, B6JRj mice showed an increase in norepinephrine levels, while in B6JCrl mice plasma norepinephrine did not differ between age groups (Fig. 2f). NT-proBNP plasma levels showed no significant differences between the two age groups (Fig. 2g).

**Table 1:** Echocardiography measurement in B6J substrains

Parameter	B6JRj young	B6JRj old	B6JCrl young	B6JCrl old
Sample size	9	10	5	6
Body weight [g]	31.54 ± 2.0	33.32 ± 3.12	30.24 ± 0.99	34.62 ± 3.31*
Heart rate [BPM]	505.72 ± 29.56	499.95 ± 27.72	420.29 ± 72.11	390.70 ± 47.13
ESV [µl]	37.02 ± 14.26	44.07 ± 12.24	28.90 ± 4.37	23.49 ± 4.43
EDV [µl]	85.52 ± 18.76	80.64 ± 13.17	66.80 ± 4.02	58.23 ± 7.56*
Ejection fraction [%]	57.86 ± 7.65	48.64 ± 3.91*	56.84 ± 5.01	59.98 ± 3.39
Stroke volume [µl]	48.50 ± 5.78	36.57 ± 10.17*	37.90 ± 3.32	34.74 ± 3.58
Cardiac output [ml/min]	24.47 ± 2.98	18.22 ± 5.00*	14.56 ± 1.42	13.48 ± 1.08
LV mass [mg]	96.94 ± 20.82	134.53 ± 18.86*	136.45 ± 30.17	140.99 ± 14.99
LV diameter s [mm]	3.03 ± 0.43	3.03 ± 0.38	2.85 ± 0.10	2.57 ± 0.44
LV diameter d [mm]	4.52 ± 0.36	4.41 ± 0.34	4.18 ± 0.16	4.07 ± 0.37
LVAW s [mm]	0.82 ± 0.07	1.04 ± 0.09*	1.34 ± 0.29	1.51 ± 0.16
LVAW d [mm]	0.59 ± 0.05	0.81 ± 0.06*	0.90 ± 0.19	0.99 ± 0.10
LVPW s [mm]	0.82 ± 0.07	1.01 ± 0.05*	1.13 ± 0.10	1.24 ± 0.20
LVPW d [mm]	0.57 ± 0.04	0.75 ± 0.06*	0.78 ± 0.06	0.81 ± 0.16

^*^p ≤ 0.05 compared within substrains using unpaired *t*-test (normality test passed) or Mann–Whitney *U*-test (normality test not passed)

*B6* C57BL/6, *d* diastole, *ESV* end-systolic volume, *EDV* end-diastolic volume, *LV* left ventricular, *LVAW* left ventricular anterior wall, *LVPW* left ventricular posterior wall, *s* systole

### Proteomic approach

**Fig. 3:**
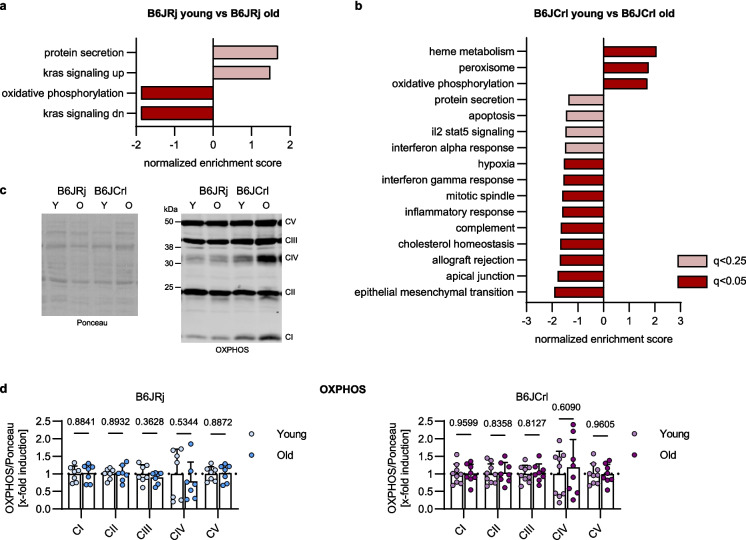
Proteomics revealed age-related differences in the cardiac oxidative phosphorylation pathway between B6JRj and B6JCrl mice. **a**, **b** Gene-set enrichment analysis (GSEA) was performed with heart proteomics data. Only cardiac relevant pathways with a *q*-value lower than 0.25 are presented (*n* = 3–4). **a** Young B6JRj mice are compared to old B6JRj mice. **b** Young B6JCrl mice are compared to old B6JCrl mice. **c**, **d** Western blot was done using OXPHOS antibody cocktail to stain for mitochondrial complex I-V, signal was normalized to total lane protein using Ponceau S staining. Differences in protein levels are demonstrated as changes compared to the young group (*n* = 7–9). Statistical significance was tested with unpaired *t*-test (normality passed) or Mann–Whitney *U* test (normality not passed) and *p*-values are shown. B6, C57BL/6; C, complex. Max, maximal; O, old; Y, young OXPHOS, oxidative phosphorylation

We next performed cardiac proteome data analysis by LC–MS/MS to investigate whether a differential protein profile between the two substrains may be linked to the differences in functional cardiac aging phenotype. Gene set enrichment analysis (GSEA) was performed by using the mouse-ortholog hallmark gene sets provided by Molecular Signatures Database (MSigDB) [23–25]. When comparing the hearts of the young groups from both substrains, more enriched cholesterol homeostasis, less enriched adipogenesis, and less enriched OXPHOS pathway were detected in young B6JRj compared to young B6JCrl mice, with OXPHOS showing the strongest discrepancies based on the normalized enrichment score (Supplements, Fig. 2). Focusing on the OXPHOS pathway, comparison of B6JRj young and B6JRj old mice revealed less enriched OXPHOS in young compared to old mice (Fig. 3a). Contrary, in B6JCrl mice, the OXPHOS pathway was more enriched in young compared to old B6JCrl mice (Fig. 3b). Western blot for mitochondrial complexes was performed to measure whether the general abundance of the complexes differs in aging. Yet, protein levels of the complex I subunit NDUFB8, complex II subunit SDHB, complex III subunit UQCRC2, complex IV subunit MTCO1, and complex V subunit ATP5A did not show age-related changes in B6JRj nor in B6JCrl mice (Fig. 3c–d). To further investigate the metabolic changes in the heart of both substrains during aging, the kinetic model CARDIOKIN1 was used. Based on the proteomic profile, maximal cardiac metabolic capacities were assessed as described in Berndt et al. [26]. Results show that maximal ATP production capacity was not affected during aging in both substrains (Table 2). Accordingly, O_2_ consumption and the ATP/O_2_ ratio were also not changed with age. However, when directly comparing the old groups of both substrains, B6JRj mice demonstrated lower maximal ATP production capacity and lower O_2_ consumption and ATP/O_2_ ratio than B6JCrl mice (*p* = 0.03, *p* = 0.04, *p* = 0.03 for B6JRj old vs B6JCrl old; Table 2).

**Table 2:** Differences in age-associated metabolic changes in two B6J substrains revealed by mathematical modulation

Parameter	B6JRj young	B6JRj old	B6JCrl young	B6JCrl old
Sample size	4	4	3	4
Max ATP production capacity [µmol/g/h]	1682 ± 327.2	954.6 ± 708.1	2039 ± 177.4	2013 ± 85.54†
Max O_2_ consumption [µmol/g/h]	461.2 ± 97.8	287.6 ± 172.1	533.6 ± 37.5	509.2 ± 25.6†
Max ATP/O_2_ ratio [µmol/g/h]	3.67 ± 0.31	3.04 ± 0.66	3.82 ± 0.07	3.96 ± 0.11†

The CARDIOKIN1 mathematical modulation was performed using heart proteomics data. Results for overnight fasting conditions are presented. *p ≤ 0.05 compared within substrains, †*p* ≤ 0.05 compared within age groups using unpaired *t*-test (normality test passed) or Mann–Whitney *U*-test (normality test not passed)

### Plasma

**Fig. 4:**
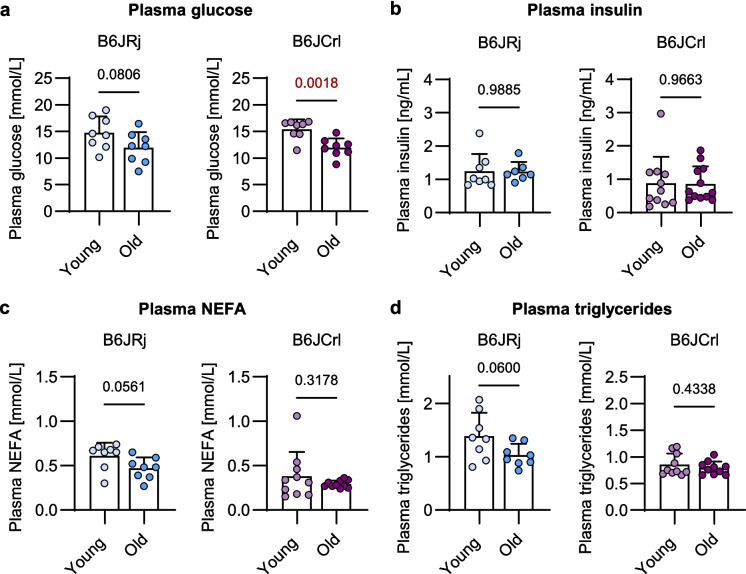
Random plasma metabolites show slight changes during aging in both substrains. **a**, **b**, **c**, **d** Glucose, insulin, non-esterified fatty acids (NEFAs), and triglycerides were measured in plasma samples of young and old mice from both substrains (*n* = 8–12). Statistical significance was tested with unpaired *t*-test (normality passed) or Mann–Whitney *U* test (normality not passed) and *p*-values are shown. B6, C57BL/6

To analyze the role of substrate availability for the aging hearts, we measured random glucose, insulin, and non-esterified fatty acids (NEFAs) as well as triglycerides in murine plasma samples. Results revealed lower plasma glucose in old compared to young B6JCrl mice; B6JRj mice showed a similar aging trend (*p* = 0.08; Fig. 4a). The random plasma insulin did not differ between the age groups of both substrains (Fig. 4b). Interestingly, for NEFAs and triglyceride levels, we observed a trend towards a reduction in aging but only in the B6JRj (*p* = 0.06 for NEFA, *p* = 0.06 for triglycerides) and not in the B6JCrl mice (Fig. 4c–d).

### Cardiomyocyte function

**Fig. 5:**
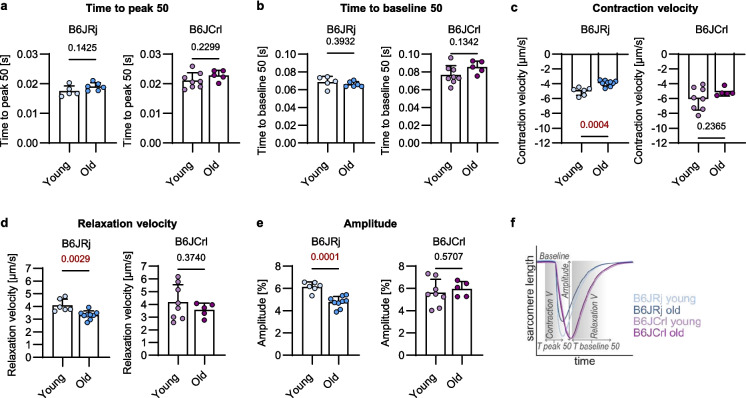
Cardiomyocyte contraction was impaired during aging in B6JRj but not in B6JCrl mice. **a**, **b**, **c, d, e **Cardiomyocyte function was analyzed directly after cardiomyocyte isolation using the IonOptix system. Contraction was measured during pacing from at least 10 cells per sample with at least 10 transients per cell (n = 5–9). **f** Results are presented in a scheme showing the changes in sarcomere length per time. Statistical significance was tested with unpaired *t*-test (normality passed) or Mann–Whitney *U* test (normality not passed) and *p*-values are shown. B6, C57BL/6; V, velocity; T, time

Finally, we investigate whether the age-related impairment of LV function is also reflected in reduced cardiomyocyte contractility, measured with the Contractility System from IonOptix. Time to peak 50 and time to baseline 50 did not change with age in both substrains (Fig. 5a, b). With age, relaxation and contraction velocity declined in B6JRj; however, B6JCrl mice did not show significant changes in contraction nor relaxation velocity (Fig. 5c–d). In accordance, the contraction amplitude of B6JRj cardiomyocytes decreased during aging, while it was not affected in B6JCrl cells (Fig. 5e). Cell contractility results showed that the reduced heart function during aging in B6JRj mice is mirrored at the cardiomyocyte level (Fig. 5f).

## Discussion

In this study, we highlight differences in the cardiac aging phenotype between two B6J substrains distributed by two vendors, B6JRj mice obtained from Janvier and B6JCrl mice obtained from Charles River. Our results identify a general age-associated impairment of systolic function in B6JRj but not in B6JCrl mice. Proteomic data indicated different age-related changes in the energy metabolism between the two substrains. This age-associated impairment of the B6JRj heart was also reflected at the cardiomyocyte functional level, as isolated cardiomyocytes from old B6JRj mice showed lower contraction and relaxation velocities as well as a reduced contraction amplitude compared to young B6JRj mice.

The animal strains used herein are common models to study aging and metabolic health [18]. According to a Japanese survey, B6J mice are 49% the most used mouse model for genetic modification. Both our B6J substrains share the same genetic background, but the B6JRjs were introduced to by Janvier in the 1990s [8]. Such a separation results in phenotypical changes, which are commonly attributed to a genetic drift caused by single nucleotide polymorphism [11, 31]. In line with this notion, Heiker et al. (2014) showed that B6JRj mice express the same rs13477019 allele as B6N mice, which however differs from B6J mice [18]. A recent study from Siersbæk et al. (2020) demonstrated that when comparing young male B6JRj and B6JCrl mice, B6JRj mice are more prone to diet-induced obesity, since they gained a higher percentage of fat after receiving high fat diet for 10 weeks compared to B6JCrl mice [13]. In the present study, we show that both substrains differ fundamentally in their cardiac aging phenotype, particularly in cardiac function. Our GSEA data comparing young hearts from B6JRj and B6JCrl revealed lower enrichment of the adipogenesis pathway in B6JRj. As such, our data support the results from Siersbæk et al. (2020) [13], but, more importantly, demonstrate to our knowledge for the first time age-related functional differences in the hearts of two B6J substrains.

Aging is associated with declining heart function. Structural changes such as LV hypertrophy or fibrosis impair contractility, often resulting in diastolic dysfunction [2]. In old B6JRj mice, heart weight and LV mass (both normalized to BW) and systolic and diastolic LVAW and LVPW thickness increased with age, which was not detectable in B6JCrl mice. Based on these results, we conclude that specifically B6JRj mice develop LV hypertrophy during aging. LV mass increases during aging; however, LV hypertrophy is not present in healthy aging [2]. LV hypertrophy evident as increased LV mass was associated with all-cause mortality in patients suffering from HF with preserved EF (HFpEF), a form of HF in which the EF is still above 50% [32]. Accordingly, we propose that the increase in LV mass in the old B6JRj mice can be related to an overall lower health status and increased mortality in this group. To study fibrosis, we measured total collagen (types I and III) levels as the main component of extracellular matrix in the heart [33]. Total collagen levels increased with age in both B6J substrains, thereby pointing towards an age-related increase in myocardial matrix deposition in old hearts in general. Especially, the deposition of extracellular matrix around the arteries was elevated with age. This increase in perivascular fibrosis indicates vascular stiffness, which can be associated with a hypertensive state and endothelial injury in the heart [34]. Fibrosis in the context of hypertrophic cardiomyopathy is an early event before hypertrophy is induced [35] which leads to the hypothesis that B6JCrl mice may exhibit a delayed cardiac aging phenotype compared to B6JRj. More precisely, the deposition in extracellular matrix began in old mice of both substrains, while the potentially subsequent increase in hypertrophy and associated cardiac impairment occurred only in the old B6JRj mice. Since we did not perform longevity analyses (> 24 month), we cannot rule out that with further aging, B6JCrl mice show a similar cardiac aging phenotype as B6JRj mice.

The increase in fibrosis indicates enhanced inflammation with age in both substrains. To analyze the role of immune cells in mediating the age-related fibrosis, we determined the percentage of CD45-positive nuclei as a marker for leukocyte infiltration. While B6JRj mice showed an increased percentage of CD45-positive cells with age, the number in B6JCrl mice did not differ. In a previous study, Trial et al. (2017) showed that in contrast to B6N mice, B6J mice from Jackson accumulate CD45-positive cells with age. However, Trial et al. (2017) used mice with an age of 3 and 20–30 months, potentially explaining the contrasting results [36]. It is known that immune cell infiltration is strongly associated with fibrosis. Especially, M2a macrophages play a crucial role in promoting extracellular matrix. Their number correlates with the degree of fibrosis [36]. However, recruitment of CD45-positive cells may not be necessary for age-related fibrosis, since the production of extracellular matrix is mediated by fibroblasts/myofibroblasts [37]. In contrast, we suggest that the recruitment of CD-45 positive cells, not the persistent number of CD45-positive cells, is a hint for cardiac dysfunction, since the increase in leukocytes indicates a reaction to inflammation or injury such as seen, e.g. in myocardial infarction [37]. Trial et al. (2017) indicated a connection of leukocyte recruitment and diastolic dysfunction in monocyte chemoattractant protein-1 knockout mice. These mice showed a reduced age-associated leukocyte recruitment and fibrosis and, interestingly, the knockout mice developed a less strong diastolic dysfunction in aging than old control mice [36].

To analyze the cardiac function and the development of HF, we performed echocardiographic analyses. B6JRj mice lack age-associated effects on EDV and ESV; however, cardiac output and stroke volume decreased with age, which also resulted in a reduced EF. Contrary in B6JCrl mice, EDV was reduced with age, and a similar trend was observed for ESV (*p* = 0.07); however, stroke volume, cardiac output, and EF were unaltered. Zhang et al. (2021) showed that by 2D, EDV and ESV decrease in old mice, comparing 2- to 24-month-old wild-type mice; however, stroke volume was not affected [16]. Roh et al. (2020) detected no differences in the EF when comparing 3–4, 24–26, and 28–30-month-old male B6J mice, yet exercise capacity declined with age in these mice [17]. Comparing these results, we suggest that these differences in the cardiac aging phenotype are attributed to the different substrains used in the studies, which again underlines the phenotypic diversity of mice substrains. During human aging, EF is largely preserved, and only declines during exercise in the elderly [2]. In our B6JRj mice, echocardiography analysis revealed HF with mildly reduced EF (HFmrEF) in the old group, since EF decreased to 48.6% [38]. HFmrEF is defined as the intermediate between HFpEF, the predominant HF type in human aging [39], and HF with reduced EF (HFrEF); although Chen et al. (2019) demonstrated that in terms of aging and all-cause mortality, HFmrEF was more similar to HFpEF than HFrEF [40]. Taken together, while B6JRj mice showed a declining systolic heart function with age, B6JCrl did not exhibit age-related cardiac functional changes. Discrepancies in heart structure and function between two B6 substrains were found in other recent studies. Williams et al. (2020) compared B6NTac with B6J mice and determined lower EDV and ESV in B6J mice together with higher LVAW and LVPW thickness and a higher EF [41]. Moreover, Zhou et al. (2021) observed no differences in the EF comparing B6J and B6NCrl mice. However, myocardial infarction caused more severe outcomes in B6NCrl than in B6J mice measured as smaller infarct size and lower mortality in B6J mice [42]. In addition to our echocardiography results, the presented studies [41, 42] produced differing results with regard to the cardiac phenotype in mice, highlighting the importance of choosing the suitable animal model.

To interpret the changes in EF in the context of HF, the plasma HF marker norepinephrine and NT-proBNP were measured. We detected an increase in norepinephrine in B6JRj but not in B6JCrl mice during aging, while NT-proBNP did not differ between the age groups. Plasma norepinephrine levels are associated with LV dysfunction in patients suffering from congestive HF [43]. Additionally, high norepinephrine levels are associated with all-cause mortality in patients with asymptomatic LV dysfunction measured by an EF of ≤ 35% [5]. However, Esler et al. (1995) pointed out that plasma norepinephrine levels also rise with age when comparing 20–30- to 60–75-year-old healthy men [44]. As such, the increase in plasma norepinephrine in B6JRj mice during aging can be associated with HF or interpreted as a distinctive change during the aging process, which was not present in B6JCrl mice. In contrast to norepinephrine, NT-proBNP is used for the clinical diagnosis of HF since elevated levels are associated with a higher risk for cardiovascular and all-cause death. Levels rise in acute HF but can also be elevated for a longer time period [45]. However, we were not able to detect differences in NT-proBNP plasma levels between the two age groups in both substrains. Yet, two mice in the old B6JRj group and one mouse in the old B6JCrl group had NT-proBNP levels above 1500 pg/ml (outlier, data not shown), which may indicate an acute HF in those mice. As such, even though mean NT-proBNP levels were not significantly increased, results may show a hint for HF in single old but not young mice of both substrains.

The dissimilarities in the cardiac aging phenotype between two substrains raised further questions on how the hearts in the two substrains may differ at the molecular level. By applying GSEA using cardiac proteome data from all groups, we identified that enrichment of the OXPHOS pathway differs during aging between the substrains. In B6JRj mice, the OXPHOS pathway was more enriched in the old group, while in B6JCrl mice, this pathway was more enriched in the young group. To exclude that the general abundance of one or more of the mitochondrial complexes differs with age, we measured protein levels using OXPHOS antibody. Results showed no differences in the protein levels of complex I, II, III, IV, and V with age. However, it is well established that cardiac aging is associated with the impairment of mitochondrial function leading to an energy deficit [46]. Notably, mitochondrial function can be impaired, even if complex protein levels remain stable [47]. Therefore, and because the GSEA revealed differences in the OXPHOS pathway enrichment, calculation of the maximal ATP production using CARDIOKIN1 modulation based on proteomic data was performed. Mathematical modulation showed no significant changes during aging in both substrains. Nevertheless, direct comparison of the two old groups revealed lower maximal ATP production, O_2_ consumption, and ATP/O_2_ ratio in B6JRj mice than B6JCrl mice, indicating differences in mitochondrial function. Studies comparing failing hearts with healthy controls demonstrated 20–30% lower ATP levels [48]. Accordingly, we propose that the lower maximal ATP production in the old B6JRj mice might be associated with the reduced systolic function in this group. One reason for the reduced maximal ATP production might be the substrate availability. Therefore, we measured random plasma metabolites including glucose, insulin, NEFAs, and triglycerides. While glucose showed the same trend in reduction during aging in both substrains, NEFAs and triglycerides appear to be only lower in the B6JRj mice with age. The heart predominantly uses fatty acids as a substrate; the second most frequently used energy substrate is glucose [7]. Thus, a reduction in available NEFAs and triglycerides and potentially also glucose may be a hint for an impaired energy production capacity and associated reduced cardiac function measured in the aging B6JRj mice.

A reduction in ATP levels due to iron deficiency has previously been shown to be associated with a reduction in cardiomyocyte function measured as a lower contraction amplitude [49]. Analogously, we observed a declined contraction amplitude with age in B6JRj mice. In these mice, contraction and relaxation velocities of the cells were reduced during aging. In contrary, in B6JCrl mice, no changes in cardiomyocyte contraction during aging were detectable. Previous studies show that the contraction amplitude is reduced during aging in rodents, even if individual studies showed no alteration [50]. This discrepancy among the studies may be at least partially explained by the use of different mouse strains. For example, in B6SJLF1/J mice [51] and B6 mice from Charles River [52], contraction amplitude declined, while in not further described B6 mice [53], the amplitude was not affected during aging.

Collectively, our present study has limitations. First, sample size is low, especially for the proteomic approach, and it differs between the substrains, although age groups within each substrain had the same size throughout the experiments. Second, while the whole group of mice did not show changes in the BW during aging, the BW of B6JCrl mice used for echocardiography increased with age. However, since BW did not correlate with stroke volume or cardiac output, we ruled out that differences in BW between B6JCrl mice and B6JCrl may be masking the age-related reduction of functional readouts. Additionally, the heart rate differed between the mice from the two substrains, which may be attributable to day-to-day and experimenter variations; nevertheless, age groups within each substrain were evaluated at the same day by the same experimenter. Lastly, even though our study indicates a systolic impairment in old B6JRj mice, further analyses of, e.g. physical performance is needed to verify the development of HF syndrome.

In summary, our data point out distinct differences in the cardiac aging phenotype of two B6J substrains. The data highlight the importance of a detailed description and categorization of the used animal model. A report by the journal diabetes showed that in the years from 2010 to 2014, 58.5% of the publications in this journal did not include complete explanations of the strain background [54]. By giving a precise description of the used mouse strain, reproducibility, which is a well-known problem in various fields of research [55], can be improved in animal studies [56]. Additionally, we elucidated the importance of choosing the suitable animal model for a new study design. While B6JRj mice showed a hypertrophic fibrotic heart with impaired functional parameters at the whole heart and cardiomyocyte level, probably caused by changes in the OXPHOS pathway, B6JCrl mice did not show cardiac dysfunction with age besides myocardial fibrosis. As such, our data reveal that compared to male B6JCrl mice, male B6JRj mice are a better model for studying age-related cardiac impairment, evident as a moderate systolic dysfunction with age. Using such interstrain comparison may be a valuable tool to identify pathways of aging versus resilience in the future.

## Supplementary Information

Below is the link to the electronic supplementary material.

Supplementary file1 (DOCX 44 KB)

Supplementary file2 (PDF 330 KB)

Supplementary file3 (PDF 326 KB)

Supplementary file4 (PDF 291 KB)

## Data Availability

All data used within this study are available upon request by the corresponding author.

## Abbreviations

B6: C57BL/6
BW: Body weight
CVD: Cardiovascular disease
EDV: End-diastolic volume
EF: Ejection fraction
ESV: End-systolic volume
FDR: False discovery rate
GSEA: Gene set enrichment analysis
HF: Heart failure
HFmrEF: Heart failure with mildly reduced ejection fraction
HFpEF: Heart failure with preserved ejection fraction
HFrEF: Heart failure with reduced ejection fraction
LV: Left ventricular
LVPW: Left ventricular posterior wall
LVAW: Left ventricular anterior wall
MSigDB: Molecular signature database
NT-proBNP: N-terminal pro B-type natriuretic peptide
OXPHOS: Oxidative phosphorylation
