# Supplementary material for: Comparative phenotyping of C57BL/6J substrains reveals distinctive patterns of cardiac aging
Source: GeroScience. 2025 Jan 30;47(3):4795–812. doi: 10.1007/s11357-025-01543-7 (PMC12181499; doi:10.1007/s11357-025-01543-7)
Supplement: Supplementary file 1 — Supplementary file1 (DOCX 44 KB) [file 11357_2025_1543_MOESM1_ESM.docx]

**Supplements Figure 1**

**a**

**b**

**Supplements Figure 1: Stroke volume and cardiac output of old B6JCrl mice do not correlate with body weight.** Correlation analyses of body weight and stroke volume as well as cardiac output and body weight were performed and 95% confidence intervals are presented. n=5-9. B6: C57BL/6.

**Supplements Figure 2**

**a**

**b**

**Supplements Figure 2: Enrichment analysis comparing hearts of young mice revealed distinct differences in the oxidative phosphorylation pathway.** Only pathways with a q-value lower than 0.25 are presented. B6: C57BL/6.
