# Supplementary material for: Comparative phenotyping of C57BL/6J substrains reveals distinctive patterns of cardiac aging
Source: GeroScience. 2025 Jan 30;47(3):4795–812. doi: 10.1007/s11357-025-01543-7 (PMC12181499; doi:10.1007/s11357-025-01543-7)
Supplement: Supplementary file 2 — Supplementary file2 (PDF 330 KB) [file 11357_2025_1543_MOESM2_ESM.pdf]

Background check, full

Sample 1 m

B6JCrl mouse, male

Sample receipt: 19.09.2024
Report Date: 24.11.2024

STR Marker Data				Analytical Results		Reference 1		Percent	Reference 2		Percent
				Probe 1 m		Jackson J		93.18%	Janvier Rj		93.43%
	Marker	Chr	Pos (Mbp)	S1, A1	S1, A2	R1, A1	R1, A2	S1-R1	R2, A1	R2, A2	S1-R2
1	D1S113	Chr1	20.74	19	19	19	19	1	19	19	1
2	D1S206	Chr1	32.93	24	24	24	24	1	24	24	1
3	D1S221	Chr1	60.97	32.2	32.2	32.2	32.2	1	32.2	32.2	1
4	D1S223	Chr1	61.53	22.2	22.2	22.2	22.2	1	22.2	22.2	1
5	D1S301	Chr1	75.2	24	24	24	24	1	24	24	1
6	D1S311	Chr1	81.59	21	21	21	21	1	21	21	1
7	D1S408	Chr1	94.21	19	19	19	19	1	19	19	1
8	D1S415	Chr1	102.87	55.2	55.2	55.2	55.2	1	55.2	55.2	1
9	D1S419	Chr1	114.91	26	26	26	26	1	26	26	1
10	D1S426	Chr1	121.86	22	22	22	22	1	22	22	1
11	D1S437	Chr1	135.57	19	19	19	19	1	19	19	1
12	D1S438	Chr1	135.58	19	19	19	19	1	19	19	1
13	D1S448	Chr1	153.63	18	19	19	19	0.75	19	19	0.75
14	D1S501	Chr1	161.01	17	17	17	17	1	17	17	1
15	D1S506	Chr1	171.22	21	21	21	21	1	21	21	1
16	D1S509	Chr1	181.77	35	35	35	35	1	35	35	1
17	D1S703	Chr1	186.74	22.3	22.3	22.3	22.3	1	22.3	22.3	1
18	D1S706	Chr1	190.02	24	24	24	24	1	24	24	1
1	D2S101	Chr2	3.05	25	25	25	25	1	25	25	1
2	D2S105	Chr2	8.37	18	18	18	18	1	18	18	1
3	D2S203	Chr2	26.66	21.2	21.2	21.2	21.2	1	21.2	21.2	1
4	D2S209	Chr2	33.42	20	20	20	20	1	20	20	1
5	D2S217	Chr2	43	21	21	21	21	1	21	21	1
6	D2S223	Chr2	47.95	18	18	18	18	1	18	18	1
7	D2S304	Chr1	64.55	23	23	23	23	1	23	23	1
8	D2S386	Chr2	79.91	25	25	25	25	1	25	25	1

GVG Diagnostics

STR Marker Data				Analytical Results		Reference 1		Percent	Reference 2		Percent
				Probe 1 m		Jackson J		93.18%	Janvier Rj		93.43%
	Marker	Chr	Pos (Mbp)	S1, A1	S1, A2	R1, A1	R1, A2	S1-R1	R2, A1	R2, A2	S1-R2
9	D2S333	Chr2	96.72	24.2	24.2	24.2	24.2	1	24.2	24.2	1
10	D2S396	Chr2	111.73	16	16	16	16	1	16	16	1
11	D2S340	Chr2	114.94	24	24	24	24	1	24	24	1
12	D2S3103	Chr2	120.43	16	16	16	16	1	16	16	1
13	D2S355	Chr2	132.28	14	14	14	14	1	14	14	1
14	D2S368	Chr2	146.38	22	22	22	22	1	22	22	1
15	D2S382	Chr2	167.14	16	16	16	16	1	16	16	1
1	D3S102	Chr3	7.7	27.2	27.2	27.2	27.2	1	27.2	27.2	1
2	D3S232	Chr3	24.91	14	14	14	14	1	14	14	1
3	D3S211	Chr3	29.22	19	19	19	19	1	19	19	1
4	D3S226	Chr3	40.07	20	20	20	20	1	20	20	1
5	D3S404	Chr3	44.49	58	58	58	58	1	58	58	1
6	D3S416	Chr3	65.9	18	18	18	18	1	18	18	1
7	D3S424	Chr3	72.96	23	23	22	22	0	22	22	0
8	D3S436	Chr3	81.7	18	18	18	18	1	18	18	1
9	D3S447	Chr3	95.22	18	18	18	18	1	18	18	1
10	D3S462	Chr3	107.22	16	16	16	16	1	16	16	1
11	D3S476	Chr3	118,62	22	22	22	22	1	22	22	1
12	D3S490	Chr3	127.96	18	18	18	18	1	18	18	1
13	D3S4103	Chr3	136.85	30	30	30	30	1	30	30	1
14	D3S4111	Chr3	147.13	17	17	17	17	1	17	17	1
15	D3S4117	Chr3	158.21	22	22	22	22	1	22	22	1
1	D4S110	Chr4	12.41	18	18	18	18	1	18	18	1
2	D4S111	Chr4	13.54	18	18	18	18	1	18	18	1
3	D4S118	Chr4	27.25	17	17	17	17	1	17	17	1
4	D4S124	Chr4	39.64	21	21	21	21	1	21	21	1
5	D4S140	Chr4	65.34	17	17	17	17	1	17	17	1
6	D4S152	Chr4	81.8	15	15	15	15	1	15	15	1
7	D4S202	Chr4	84.49	24	24	24	24	1	24	24	1
8	D4S216	Chr4	114.61	17	17	17	17	1	17	17	1
9	D4S305	Chr4	135.37	16	16	16	16	1	16	16	1
10	D4S309	Chr4	141.09	25.2	25.2	25.2	25.2	1	25.2	25.2	1
11	D4S317	Chr4	155.56	20	20	20	20	1	20	20	1
1	D5S219	Chr5	20.91	16	16	16	16	1	16	16	1
2	D5S303	Chr5	34.6	18	19	19	19	0.75	19	19	0.75
3	D5S314	Chr5	49.76	29	29	29	29	1	29	29	1
4	D5S326	Chr5	59.51	20	20	20	20	1	20	20	1
5	D5S403	Chr5	74.17	20	20	20	20	1	20	20	1
6	D5S509	Chr5	88.9	29	30	29	29	0.75	29	29	0.75
7	D5S517	Chr5	99.84	19	19	19	19	1	19	19	1
8	D5S519	Chr5	108.46	19	20	19	20	1	19	19	0.75

GVG Diagnostics

STR Marker Data				Analytical Results		Reference 1		Percent	Reference 2		Percent
				Probe 1 m		Jackson J		93.18%	Janvier Rj		93.43%
	Marker	Chr	Pos (Mbp)	S1, A1	S1, A2	R1, A1	R1, A2	S1-R1	R2, A1	R2, A2	S1-R2
9	D5S602	Chr5	117.56	23	23	23	23	1	23	23	1
10	D5S703	Chr5	127.31	20	20	20	20	1	20	20	1
11	D5S811	Chr5	135.58	18	18	18	18	1	18	18	1
1	D6S103	Chr6	15.39	21	21	21	21	1	21	21	1
2	D6S109	Chr6	23.41	20	20	20	20	1	20	20	1
3	D6S203	Chr6	38.65	17	18	17	17	0.75	17	17	0.75
4	D6S209	Chr6	47.59	19	19	19	19	1	19	19	1
5	D6S215	Chr6	58.87	25	25	25	25	1	25	25	1
6	D6S221	Chr6	72.19	20	20	20	20	1	20	20	1
7	D6S224	Chr6	74.82	18	18	18	18	1	18	18	1
8	D6S230	Chr6	91.83	14	14	16	16	0	16	16	0
9	D6S241	Chr6	100.74	24	25	24	24	0.75	24	24	0.75
10	D6S246	Chr6	105.22	23	24	23	23	0.75	23	23	0.75
11	D6S280	Chr6	110.19	40	40	40	40	1	40	40	1
12	D6S253	Chr6	120.38	18	18	15	15	0	15	15	0
13	D6S266	Chr6	134.95	24	24	24	24	1	24	24	1
14	D6S401	Chr6	148.78	32	33	32	32	0.75	32	32	1
1	D7S102	Chr7	3.22	22	22	22	22	1	22	22	1
2	D7S107	Chr7	17.05	17	17	17	17	1	17	17	1
3	D7S123	Chr7	30.67	20	20	20	20	1	20	20	1
4	D7S113	Chr7	30.82	15	16	18	18	0	17	17	0
5	D7S204	Chr7	46.09	26	26	26	26	1	26	26	1
6	D7S210	Chr7	61.13	43	43	43	43	1	43	43	1
7	D7S222	Chr7	76.48	19	19	19	19	1	19	19	1
8	D7S303	Chr7	90.2	21	21	21	21	1	21	21	1
9	D7S305	Chr7	92	17	17	17	17	1	17	17	1
10	D7S320	Chr7	105.53	19	19	19	19	1	19	19	1
11	D7S326	Chr7	113.61	19	19	19	19	1	19	19	1
12	D7S335	Chr7	128.73	19	19	19	19	1	19	19	1
13	D7S501	Chr7	144.02	23	23	23	23	1	23	23	
1	D8S105	Chr8	8.61	19	20	19	19	0.75	20	20	0.75
2	D8S202	Chr8	23.77	23	23	23	23	1	23	23	1
3	D8S209	Chr8	30.51	14	14	14	14	1	14	14	1
4	D8S302	Chr8	42.09	22	22	22	22	1	22	22	1
5	D8S304	Chr8	44.65	15	15	15	15	1	15	15	1
6	D8S313	Chr8	61.18	24	24	24	24	1	24	24	1
7	D8S504	Chr8	80.12	19	19	19	19	1	19	19	1
8	D8S516	Chr8	92.14	21	21	21	21	1	21	21	1
9	D8S529	Chr8	106.4	19	19	19	19	1	19	19	1
10	D8S539	Chr8	115.01	22	22	22	22	1	22	22	1
11	D8S542	Chr8	118.06	20.3	20.3	20.3	20.3	1	20.3	20.3	1

GVG Diagnostics

STR Marker Data				Analytical Results		Reference 1		Percent	Reference 2		Percent
				Probe 1 m		Jackson J		93.18%	Janvier Rj		93.43%
	Marker	Chr	Pos (Mbp)	S1, A1	S1, A2	R1, A1	R1, A2	S1-R1	R2, A1	R2, A2	S1-R2
12	D8S554	Chr8	127.97	21.2	22.2	21.2	21.2	0.75	21.2	21.2	0.75
1	D9S201	Chr9	14.69	13	14	14	14	0.75	14	14	1
2	D9S204	Chr9	21,45	18	18	18	18	1	18	18	1
3	D9S211	Chr9	38.19	18	18	18	18	1	18	18	1
4	D9S213	Chr9	37.53	23	23	23	23	1	23	23	1
5	D9S218	Chr9	46.44	18	19	18	18	0.75	18	18	0.75
6	D9S219	Chr9	47.17	25.2	25.2	25.2	25.2	1	25.2	25.2	1
7	D9S304	Chr9	64.81	12	12	12	12	1	12	12	1
8	D9S308	Chr9	69.95	21	21	21	21	1	21	21	1
9	D9S312	Chr9	80.21	19	19	19	19	1	19	19	1
10	D9S314	Chr9	82.76	23	23	23	23	1	23	23	1
11	D9S323	Chr9	100.54	24	24	24	24	1	24	24	1
12	D9S410	Chr9	107.86	11	12	13	13	0	13	13	0
13	D9S511	Chr9	108.96	20	20	20	20	1	20	20	1
14	D9S516	Chr9	108.96	18	18	18	18	1	18	18	1
1	D10S205	Chr10	12.91	20	20	20	20	1	20	20	1
2	D10S213	Chr10	28.11	20	20	20	20	1	20	20	1
3	D10S247	Chr10	42.91	13	13	13	13	1	13	13	1
4	D10S218	Chr10	43.39	23.2	23.2	23.2	23.2	1	23.2	23.2	1
5	D10S257	Chr10	53.78	43	43	43	43	1	43	43	1
6	D10S304	Chr10	65.19	23.2	23.2	23.2	23.2	1	23.2	23.2	1
7	D10S318	Chr10	80.37	23	23	23	23	1	23	23	1
8	D10S379	Chr10	93.67	16	16	16	16	1	16	16	1
9	D10S391	Chr10	104.79	34.3	34.3	34.3	34.3	1	34.3	34.3	1
10	D10S344	Chr10	115.16	21	21	21	21	1	21	21	1
11	D10S351	Chr10	121.31	20	20	20	20	1	20	20	1
1	D11S106	Chr11	18.75	21	21	21	21	1	20	21	1
2	D11S110	Chr11	22.19	22	22	22	22	1	22	22	1
3	D11S112	Chr11	30.7	20	20	20	20	1	19	19	0
4	D11S207	Chr11	43.93	17	17	17	17	1	17	17	1
5	D11S208	Chr11	33.97	16	16	16	16	1	16	16	1
6	D11S211	Chr11	47.44	17	17	17	17	1	17	17	1
7	D11S219	Chr11	60.91	14	17	17	17	0.75	17	17	0.75
8	D11S226	Chr11	75.38	16	16	16	16	1	16	16	1
9	D11S232	Chr11	89.21	20	20	20	20	1	20	20	1
10	D11S251	Chr11	105.92	24.2	24.2	24.2	24.2	1	24.2	24.2	1
11	D11S262	Chr11	120.51	11	11	11	11	1	11	11	1
1	D12S102	Chr12	8.51	21	21	21	21	1	21	22	1
2	D12S104	Chr12	10.9	20	20	20	20	1	20	20	1
3	D12S118	Chr12	37.86	50	50	50	50	1	50	50	1
4	D12S206	Chr12	54.68	24	24	21	21	0	21	21	0

GVG Diagnostics

STR Marker Data				Analytical Results		Reference 1		Percent	Reference 2		Percent
				Probe 1 m		Jackson J		93.18%	Janvier Rj		93.43%
	Marker	Chr	Pos (Mbp)	S1, A1	S1, A2	R1, A1	R1, A2	S1-R1	R2, A1	R2, A2	S1-R2
5	D12S208	Chr12	56.69	22	22	22	22	1	22	22	1
6	D12S212	Chr12	69.15	17	17	17	17	1	17	17	1
7	D12S283	Chr12	80.28	16	16	16	16	1	16	16	1
8	D12S235	Chr12	106.99	19	19	19	19	1	19	19	1
9	D12S240	Chr12	117.63	15	15	15	15	1	15	15	1
1	D13S202	Chr13	15.43	16	16	16	16	1	16	16	1
2	D13S214	Chr13	29.92	13	13	13	13	1	13	13	1
3	D13S215	Chr13	30.79	22	22	22	22	1	22	22	1
4	D13S310	Chr13	46.19	25	25	25	25	1	24	25	1
5	D13S409	Chr13	70.3	23	23	23	23	1	23	23	1
6	D13S426	Chr13	85.43	17	17	16	16	0	16	17	0.75
7	D13S501	Chr13	96.58	18	18	19	19	0	19	19	0
8	D13S516	Chr13	109.4	17	17	17	17	1	17	17	1
9	D13S524	Chr13	117.31	19	19	19	19	1	19	19	1
10	D13S525	Chr13	118.89	21	21	21	21	1	20	21	1
1	D14S108	Chr14	8.48	24	24	24	24	1	23	24	1
2	D14S204	Chr14	22.42	19	19	19	19	1	19	19	1
3	D14S260	Chr14	33.78	51	51	51	51	1	51	51	1
4	D14S291	Chr14	45.7	17	17	17	17	1	17	17	1
5	D14S213	Chr14	48.4	24	24	24	24	1	24	24	1
6	D14S269	Chr14	60.02	16	16	16	16	1	16	16	1
7	D14S224	Chr14	74.03	17	17	17	17	1	17	17	1
8	D14S237	Chr14	88.32	24	24	25	25	0	25	25	0
9	D14S274	Chr14	101.59	15	15	15	15	1	15	15	1
10	D14S283	Chr14	116.03	23	23	23	23	1	23	23	1
1	D15S209	Chr15	20.39	34.2	34.2	34.2	34.2	1	34.2	34.2	1
2	D15S215	Chr15	24.6	19	19	19	19	1	19	19	1
3	D15S217	Chr15	33.98	20	20	20	20	1	20	20	1
4	D15S219	Chr15	37.24	19	19	19	19	1	19	19	1
5	D15S304	Chr15	45.27	20	20	20	20	1	20	20	1
6	D15S314	Chr15	62.49	19	19	19	19	1	19	19	1
7	D15S324	Chr15	74.1	19	19	19	19	1	19	19	1
8	D15S336	Chr15	84.87	20	20	20	20	1	20	20	1
9	D15S345	Chr15	95.17	15	15	15	15	1	15	15	1
1	D16S109	Chr16	14.05	19	19	19	19	1	19	19	1
2	D16S120	Chr16	27.98	18	18	18	18	1	18	18	1
3	D16S124	Chr16	30.3	27	27	23	23	0	23	23	0
4	D16S137	Chr16	43.92	21.2	21.2	21.2	21.2	1	21.2	21.2	1
5	D16S147	Chr16	57.62	23	23	23	23	1	23	23	1
6	D16S204	Chr16	74.23	19	19	19	19	1	19	19	1
7	D16S221	Chr16	91.1	18	18	18	18	1	18	18	1

STR Marker Data				Analytical Results		Reference 1		Percent	Reference 2		Percent
				Probe 1 m		Jackson J		93.18%	Janvier Rj		93.43%
	Marker	Chr	Pos (Mbp)	S1, A1	S1, A2	R1, A1	R1, A2	S1-R1	R2, A1	R2, A2	S1-R2
8	D16S229	Chr16	97.29	18	18	18	18	1	18	18	1
1	D17S315	Chr17	17.48	20	21	21	21	0.75	21	21	1
2	D17S338	Chr17	31.82	20	20	20	20	1	19	20	1
3	D17S324	Chr17	41.5	17	17	17	17	1	17	17	1
4	D17S303	Chr17	57.18	19	19	17	17	0	17	17	0
5	D17S346	Chr17	70.79	13	13	13	13	1	13	13	1
6	D17S353	Chr17	79.97	17	17	17	17	1	17	17	1
7	D17S361	Chr17	88.93	22	22	22	22	1	22	22	1
1	D18S145	Chr18	3.3	21	21	21	21	1	21	21	1
2	D18S306	Chr18	3.55	58	58	58	58	1	58	58	1
3	D18S144	Chr18	16.61	19	19	19	19	1	18	19	1
4	D18S110	Chr18	17.92	15	15	15	15	1	15	15	1
5	D18S109	Chr18	29.81	15	15	15	15	1	15	15	1
6	D18S116	Chr18	43.04	17	17	17	17	1	17	17	1
7	D18S124	Chr18	44.76	29.2	29.2	29.2	29.2	1	29.2	29.2	1
8	D18S126	Chr18	62.05	16	16	16	16	1	16	16	1
9	D18S138	Chr18	67.63	20	20	20	20	1	20	20	1
10	D18S103	Chr18	69.07	15	15	15	15	1	15	15	1
11	D18S102	Chr18	83.51	39	39	39	39	1	39	39	1
1	D19S201	Chr19	8.76	16	16	16	16	1	16	16	1
2	D19S205	Chr19	16.13	17	17	17	17	1	17	17	1
3	D19S210	Chr19	33.04	21	21	21	21	1	21	21	1
4	D19S238	Chr19	49.53	20.3	20.3	20.3	20.3	1	20.3	20.3	1
5	D19S238	Chr19	53.57	20.3	20.3	20.3	20.3	1	20.3	20.3	1
1	DXS201	X	7.67	21.2		21.2		1	21.2		1
2	DXS207	X	18.46	18		18		1	18		1
3	DXS1107	X	40.66	23		23		1	23		1
4	DXS1106	X	46.64	38		38		1	38		1
5	DXS1301	X	59.07	18		18		1	18		1
6	DXS1319	X	73.53	23		23		1	22		0
7	DXS1304	X	83.42	19.2		19.2		1	19.2		1
8	DXS1308	X	96.71	15.3		15.3		1	15.3		1
9	DXS1312	X	104.29	18		18		1	18		1
10	DXS1405	X	117.87	28		28		1	28		1
11	DXS1602	X	134.74	19		19		1	19		1
12	DXS1608	X	138.09	22		22		1	22		1
13	DXS1713	X	150.95	17		17		1	17		1
14	DXS1705	X	159.41	16		16		1	16		1
15	DXS1709	X	166.49	19		19		1	19		1
1	DY101	Y	0.19	21		21		1	21		1
2	DY101I	Y	0.2	24		24		1	24		1

GVG Diagnostics

STR Marker Data				Analytical Results		Reference 1		Percent	Reference 2		Percent
				Probe 1 m		Jackson J		93.18%	Janvier Rj		93.43%
	Marker	Chr	Pos (Mbp)	S1, A1	S1, A2	R1, A1	R1, A2	S1-R1	R2, A1	R2, A2	S1-R2
3	DYS102	Y	0.33	24		24		1	24		1
4	DYS201	Y	0.94	28		28		1	28		1
5	DYS202	Y	1.73	14		14		1	14		1
6	DYS204	Y	2.57	20		20		1	20		1
7	DYS301	Y	3.99	16		16		1	16		1
8	DYS465	Y	46.6	15		15		1	15		1
9	DYS465I	Y	59.6	16		16		1	16		1
10	DYS601	Y	12.87	14		14		1	14		1
11	DYS601I	Y	17.38	16		16		1	16		1
12	DYS704	Y	17.38	18		18		1	18		1
14	DYS1001I	Y	18.38	7		7		1	7		1
13	DYS1001	Y	41.07	12		12		1	12		1
15	DYS1501	Y	81.16	11		11		1	11		1
16	DYS1501I	Y	35.69	12		12		1	12		1
17	DYS1501II	Y	36	17		17		1	17		1
