# Supplementary material for: Comparative phenotyping of C57BL/6J substrains reveals distinctive patterns of cardiac aging
Source: GeroScience. 2025 Jan 30;47(3):4795–812. doi: 10.1007/s11357-025-01543-7 (PMC12181499; doi:10.1007/s11357-025-01543-7)
Supplement: Supplementary file 3 — Supplementary file3 (PDF 326 KB) [file 11357_2025_1543_MOESM3_ESM.pdf]

GVG Diagnostics

Background check, full

Sample 4 m

B6JRj mouse, male

Sample receipt: 19.09.2024
Report Date: 24.11.2024

STR Marker Data				Analytical Results		Reference 1		Percent	Reference 2		Percent
				Probe 1 m		Jackson J		87.38%	Janvier Rj		86.92%
	Marker	Chr	Pos (Mbp)	S1, A1	S1, A2	R1, A1	R1, A2	S1-R1	R2, A1	R2, A2	S1-R2
1	D1S113	Chr1	20.74	19	19	19	19	1	19	19	1
2	D1S206	Chr1	32.93	24	24	24	24	1	24	24	1
3	D1S221	Chr1	60.97	32.2	32.2	32.2	32.2	1	32.2	32.2	1
4	D1S223	Chr1	61.53	22.2	22.2	22.2	22.2	1	22.2	22.2	1
5	D1S301	Chr1	75.2	24	24	24	24	1	24	24	1
6	D1S311	Chr1	81.59	21	21	21	21	1	21	21	1
7	D1S408	Chr1	94.21	20	20	19	19	0	19	19	0
8	D1S415	Chr1	102.87	55.2	55.2	55.2	55.2	1	55.2	55.2	1
9	D1S419	Chr1	114.91	26	26	26	26	1	26	26	1
10	D1S426	Chr1	121.86	22	22	22	22	1	22	22	1
11	D1S437	Chr1	135.57	18	18	19	19	0	19	19	0
12	D1S438	Chr1	135.58	18	18	19	19	0	19	19	0
13	D1S448	Chr1	153.63	19	19	19	19	1	19	19	1
14	D1S501	Chr1	161.01	17	17	17	17	1	17	17	1
15	D1S506	Chr1	171.22	21	21	21	21	1	21	21	1
16	D1S509	Chr1	181.77	35	35	35	35	1	35	35	1
17	D1S703	Chr1	186.74	22.3	22.3	22.3	22.3	1	22.3	22.3	1
18	D1S706	Chr1	190.02	24	24	24	24	1	24	24	1
1	D2S101	Chr2	3.05	25	25	25	25	1	25	25	1
2	D2S105	Chr2	8.37	18	18	18	18	1	18	18	1
3	D2S203	Chr2	26.66	21.2	21.2	21.2	21.2	1	21.2	21.2	1
4	D2S209	Chr2	33.42	20	20	20	20	1	20	20	1
5	D2S217	Chr2	43	21	21	21	21	1	21	21	1
6	D2S223	Chr2	47.95	18	18	18	18	1	18	18	1
7	D2S304	Chr1	64.55	23	23	23	23	1	23	23	1
8	D2S386	Chr2	79.91	25	25	25	25	1	25	25	1

STR Marker Data				Analytical Results		Reference 1		Percent	Reference 2		Percent
				Probe 1 m		Jackson J		87.38%	Janvier Rj		86.92%
	Marker	Chr	Pos (Mbp)	S1, A1	S1, A2	R1, A1	R1, A2	S1-R1	R2, A1	R2, A2	S1-R2
9	D2S333	Chr2	96.72	24.2	24.2	24.2	24.2	1	24.2	24.2	1
10	D2S396	Chr2	111.73	16	16	16	16	1	16	16	1
11	D2S340	Chr2	114.94	24	24	24	24	1	24	24	1
12	D2S3103	Chr2	120.43	16	16	16	16	1	16	16	1
13	D2S355	Chr2	132.28	14	14	14	14	1	14	14	1
14	D2S368	Chr2	146.38	21	22	22	22	0.75	22	22	0.75
15	D2S382	Chr2	167.14	16	16	16	16	1	16	16	1
1	D3S102	Chr3	7.7	27.2	27.2	27.2	27.2	1	27.2	27.2	1
2	D3S232	Chr3	24.91	14	14	14	14	1	14	14	1
3	D3S211	Chr3	29.22	19	19	19	19	1	19	19	1
4	D3S226	Chr3	40.07	20	20	20	20	1	20	20	1
5	D3S404	Chr3	44.49	58	58	58	58	1	58	58	1
6	D3S416	Chr3	65.9	18	18	18	18	1	18	18	1
7	D3S424	Chr3	72.96	22	22	22	22	1	22	22	1
8	D3S436	Chr3	81.7	18	18	18	18	1	18	18	1
9	D3S447	Chr3	95.22	18	18	18	18	1	18	18	1
10	D3S462	Chr3	107.22	16	16	16	16	1	16	16	1
11	D3S476	Chr3	118,62	22	22	22	22	1	22	22	1
12	D3S490	Chr3	127.96	18	18	18	18	1	18	18	1
13	D3S4103	Chr3	136.85	30	30	30	30	1	30	30	1
14	D3S4111	Chr3	147.13	17	17	17	17	1	17	17	1
15	D3S4117	Chr3	158.21	22	22	22	22	1	22	22	1
1	D4S110	Chr4	12.41	18	18	18	18	1	18	18	1
2	D4S111	Chr4	13.54	18	18	18	18	1	18	18	1
3	D4S118	Chr4	27.25	17	17	17	17	1	17	17	1
4	D4S124	Chr4	39.64	21	21	21	21	1	21	21	1
5	D4S140	Chr4	65.34	17	17	17	17	1	17	17	1
6	D4S152	Chr4	81.8	15	15	15	15	1	15	15	1
7	D4S202	Chr4	84.49	23	23	24	24	0	24	24	0
8	D4S216	Chr4	114.61	17	17	17	17	1	17	17	1
9	D4S305	Chr4	135.37	16	16	16	16	1	16	16	1
10	D4S309	Chr4	141.09	25.2	25.2	25.2	25.2	1	25.2	25.2	1
11	D4S317	Chr4	155.56	20	20	20	20	1	20	20	1
1	D5S219	Chr5	20.91	16	16	16	16	1	16	16	1
2	D5S303	Chr5	34.6	19	19	19	19	1	19	19	1
3	D5S314	Chr5	49.76	29	29	29	29	1	29	29	1
4	D5S326	Chr5	59.51	20	20	20	20	1	20	20	1
5	D5S403	Chr5	74.17	21	21	20	20	0	20	20	0
6	D5S509	Chr5	88.9	29	29	29	29	1	29	29	1
7	D5S517	Chr5	99.84	19	19	19	19	1	19	19	1
8	D5S519	Chr5	108.46	19	19	19	20	1	19	19	1

STR Marker Data				Analytical Results		Reference 1		Percent	Reference 2		Percent
				Probe 1 m		Jackson J		87.38%	Janvier Rj		86.92%
	Marker	Chr	Pos (Mbp)	S1, A1	S1, A2	R1, A1	R1, A2	S1-R1	R2, A1	R2, A2	S1-R2
9	D5S602	Chr5	117.56	23	23	23	23	1	23	23	1
10	D5S703	Chr5	127.31	20	20	20	20	1	20	20	1
11	D5S811	Chr5	135.58	18	18	18	18	1	18	18	1
1	D6S103	Chr6	15.39	21	21	21	21	1	21	21	1
2	D6S109	Chr6	23.41	20	20	20	20	1	20	20	1
3	D6S203	Chr6	38.65	17	17	17	17	1	17	17	1
4	D6S209	Chr6	47.59	19	19	19	19	1	19	19	1
5	D6S215	Chr6	58.87	25	25	25	25	1	25	25	1
6	D6S221	Chr6	72.19	20	20	20	20	1	20	20	1
7	D6S224	Chr6	74.82	17	17	18	18	0	18	18	0
8	D6S230	Chr6	91.83	16	16	16	16	1	16	16	1
9	D6S241	Chr6	100.74	25	25	24	24	0	1	24	0
10	D6S246	Chr6	105.22	22	23	23	23	0.75	23	23	0.75
11	D6S280	Chr6	110.19	40	40	40	40	1	40	40	1
12	D6S253	Chr6	120.38	15	15	15	15	1	15	15	1
13	D6S266	Chr6	134.95	24	24	24	24	1	24	24	1
14	D6S401	Chr6	148.78	32	32	32	32	1	32	32	1
1	D7S102	Chr7	3.22	23	23	22	22	0	22	22	0
2	D7S107	Chr7	17.05	17	18	17	17	0.75	17	17	0.75
3	D7S123	Chr7	30.67	20	20	20	20	1	20	20	1
4	D7S113	Chr7	30.82	18	18	18	18	1	17	17	1
5	D7S204	Chr7	46.09	26	26	26	26	1	26	26	1
6	D7S210	Chr7	61.13	43	43	43	43	1	43	43	1
7	D7S222	Chr7	76.48	19	19	19	19	1	19	19	1
8	D7S303	Chr7	90.2	21	21	21	21	1	21	21	1
9	D7S305	Chr7	92	17	17	17	17	1	17	17	1
10	D7S320	Chr7	105.53	19	19	19	19	1	19	19	1
11	D7S326	Chr7	113.61	19	19	19	19	1	19	19	1
12	D7S335	Chr7	128.73	19	19	19	19	1	19	19	1
13	D7S501	Chr7	144.02	22	22	23	23	0	23	23	0
1	D8S105	Chr8	8.61	19	19	19	19	1	20	20	0
2	D8S202	Chr8	23.77	23	23	23	23	1	23	23	1
3	D8S209	Chr8	30.51	14	14	14	14	1	14	14	1
4	D8S302	Chr8	42.09	22	22	22	22	1	22	22	1
5	D8S304	Chr8	44.65	15	15	15	15	1	15	15	1
6	D8S313	Chr8	61.18	24	24	24	24	1	24	24	1
7	D8S504	Chr8	80.12	19	19	19	19	1	19	19	1
8	D8S516	Chr8	92.14	20	20	21	21	0	21	21	0
9	D8S529	Chr8	106.4	19	19	19	19	1	19	19	1
10	D8S539	Chr8	115.01	22	22	22	22	1	22	22	1
11	D8S542	Chr8	118.06	20.3	20.3	20.3	20.3	1	20.3	20.3	1

GVG Diagnostics

STR Marker Data				Analytical Results		Reference 1		Percent	Reference 2		Percent
				Probe 1 m		Jackson J		87.38%	Janvier Rj		86.92%
	Marker	Chr	Pos (Mbp)	S1, A1	S1, A2	R1, A1	R1, A2	S1-R1	R2, A1	R2, A2	S1-R2
12	D8S554	Chr8	127.97	21.2	21.2	21.2	21.2	1	21.2	21.2	1
1	D9S201	Chr9	14.69	14	14	14	14	1	14	14	1
2	D9S204	Chr9	21,45	16	16	18	18	0	18	18	0
3	D9S211	Chr9	38.19	18	18	18	18	1	18	18	1
4	D9S213	Chr9	37.53	23	23	23	23	1	23	23	1
5	D9S218	Chr9	46.44	18	18	18	18	1	18	18	1
6	D9S219	Chr9	47.17	25.2	25.2	25.2	25.2	1	25.2	25.2	1
7	D9S304	Chr9	64.81	12	12	12	12	1	12	12	1
8	D9S308	Chr9	69.95	21	21	21	21	1	21	21	1
9	D9S312	Chr9	80.21	20	20	19	19	0	19	19	0
10	D9S314	Chr9	82.76	23	23	23	23	1	23	23	1
11	D9S323	Chr9	100.54	24	24	24	24	1	24	24	1
12	D9S410	Chr9	107.86	11	12	13	13	0	13	13	0
13	D9S511	Chr9	108.96	20	20	20	20	1	20	20	1
14	D9S516	Chr9	108.96	18	18	18	18	1	18	18	1
1	D10S205	Chr10	12.91	20	20	20	20	1	20	20	1
2	D10S213	Chr10	28.11	20	20	20	20	1	20	20	1
3	D10S247	Chr10	42.91	13	13	13	13	1	13	13	1
4	D10S218	Chr10	43.39	23.2	23.2	23.2	23.2	1	23.2	23.2	1
5	D10S257	Chr10	53.78	43	43	43	43	1	43	43	1
6	D10S304	Chr10	65.19	23.2	23.2	23.2	23.2	1	23.2	23.2	1
7	D10S318	Chr10	80.37	23	23	23	23	1	23	23	1
8	D10S379	Chr10	93.67	16	16	16	16	1	16	16	1
9	D10S391	Chr10	104.79	33.3	33.3	34.3	34.3	0	34.3	34.3	0
10	D10S344	Chr10	115.16	21	21	21	21	1	21	21	1
11	D10S351	Chr10	121.31	20	20	20	20	1	20	20	1
1	D11S106	Chr11	18.75	22	23	21	21	0	20	21	0
2	D11S110	Chr11	22.19	22	22	22	22	1	22	22	1
3	D11S112	Chr11	30.7	20	20	20	20	1	19	19	0
4	D11S207	Chr11	43.93	17	17	17	17	1	17	17	1
5	D11S208	Chr11	33.97	16	16	16	16	1	16	16	1
6	D11S211	Chr11	47.44	17	17	17	17	1	17	17	1
7	D11S219	Chr11	60.91	18	18	17	17	0	17	17	0
8	D11S226	Chr11	75.38	16	16	16	16	1	16	16	1
9	D11S232	Chr11	89.21	20	20	20	20	1	20	20	1
10	D11S251	Chr11	105.92	24.2	24.2	24.2	24.2	1	24.2	24.2	1
11	D11S262	Chr11	120.51	11	11	11	11	1	11	11	1
1	D12S102	Chr12	8.51	21	22	21	21	1	21	22	1
2	D12S104	Chr12	10.9	21	21	20	20	0	20	20	0
3	D12S118	Chr12	37.86	50	50	50	50	1	50	50	1
4	D12S206	Chr12	54.68	21	21	21	21	1	21	21	1

STR Marker Data				Analytical Results		Reference 1		Percent	Reference 2		Percent
				Probe 1 m		Jackson J		87.38%	Janvier Rj		86.92%
	Marker	Chr	Pos (Mbp)	S1, A1	S1, A2	R1, A1	R1, A2	S1-R1	R2, A1	R2, A2	S1-R2
5	D12S208	Chr12	56.69	22	22	22	22	1	22	22	1
6	D12S212	Chr12	69.15	17	17	17	17	1	17	17	1
7	D12S283	Chr12	80.28	16	16	16	16	1	16	16	1
8	D12S235	Chr12	106.99	19	19	19	19	1	19	19	1
9	D12S240	Chr12	117.63	15	15	15	15	1	15	15	1
1	D13S202	Chr13	15.43	16	16	16	16	1	16	16	1
2	D13S214	Chr13	29.92	13	13	13	13	1	13	13	1
3	D13S215	Chr13	30.79	22	22	22	22	1	22	22	1
4	D13S310	Chr13	46.19	25	25	25	25	1	24	25	1
5	D13S409	Chr13	70.3	24	24	23	23	0	23	23	0
6	D13S426	Chr13	85.43	17	17	16	16	0	16	17	1
7	D13S501	Chr13	96.58	18	18	19	19	0	19	19	0
8	D13S516	Chr13	109.4	17	17	17	17	1	17	17	1
9	D13S524	Chr13	117.31	19	19	19	19	1	19	19	1
10	D13S525	Chr13	118.89	21	21	20	21	1	20	21	1
1	D14S108	Chr14	8.48	24	24	23	24	1	23	24	1
2	D14S204	Chr14	22.42	19	19	19	19	1	19	19	1
3	D14S260	Chr14	33.78	51	51	51	51	1	51	51	1
4	D14S291	Chr14	45.7	17	17	17	17	1	17	17	1
5	D14S213	Chr14	48.4	22	22	24	24	0	24	24	0
6	D14S269	Chr14	60.02	16	16	16	16	1	16	16	1
7	D14S224	Chr14	74.03	17	17	17	17	1	17	17	1
8	D14S237	Chr14	88.32	24	24	25	25	0	25	25	0
9	D14S274	Chr14	101.59	15	15	15	15	1	15	15	1
10	D14S283	Chr14	116.03	23	23	23	23	1	23	23	1
1	D15S209	Chr15	20.39	34.2	34.2	34.2	34.2	1	34.2	34.2	1
2	D15S215	Chr15	24.6	19	19	19	19	1	19	19	1
3	D15S217	Chr15	33.98	20	20	20	20	1	20	20	1
4	D15S219	Chr15	37.24	19	19	19	19	1	19	19	1
5	D15S304	Chr15	45.27	20	20	20	20	1	20	20	1
6	D15S314	Chr15	62.49	19	19	19	19	1	19	19	1
7	D15S324	Chr15	74.1	19	19	19	19	1	19	19	1
8	D15S336	Chr15	84.87	20	20	20	20	1	20	20	1
9	D15S345	Chr15	95.17	15	15	15	15	1	15	15	1
1	D16S109	Chr16	14.05	19	19	19	19	1	19	19	1
2	D16S120	Chr16	27.98	17	17	18	18	0	18	18	0
3	D16S124	Chr16	30.3	27	27	23	23	0	23	23	0
4	D16S137	Chr16	43.92	21.2	21.2	21.2	21.2	1	21.2	21.2	1
5	D16S147	Chr16	57.62	22	22	23	23	0	23	23	0
6	D16S204	Chr16	74.23	19	19	19	19	1	19	19	1
7	D16S221	Chr16	91.1	18	18	18	18	1	18	18	1

STR Marker Data				Analytical Results		Reference 1		Percent	Reference 2		Percent
				Probe 1 m		Jackson J		87.38%	Janvier Rj		86.92%
	Marker	Chr	Pos (Mbp)	S1, A1	S1, A2	R1, A1	R1, A2	S1-R1	R2, A1	R2, A2	S1-R2
8	D16S229	Chr16	97.29	18	18	18	18	1	18	18	1
1	D17S315	Chr17	17.48	20	21	21	21	0.75	21	21	0.75
2	D17S338	Chr17	31.82	20	20	20	20	1	19	20	1
3	D17S324	Chr17	41.5	17	17	17	17	1	17	17	1
4	D17S303	Chr17	57.18	19	19	17	17	0	17	17	0
5	D17S346	Chr17	70.79	13	13	13	13	1	13	13	1
6	D17S353	Chr17	79.97	17	17	17	17	1	17	17	1
7	D17S361	Chr17	88.93	22	22	22	22	1	22	22	1
1	D18S145	Chr18	3.3	21	21	21	21	1	21	21	1
2	D18S306	Chr18	3.55	58	58	58	58	1	58	58	1
3	D18S144	Chr18	16.61	19	19	19	19	1	18	19	1
4	D18S110	Chr18	17.92	15	15	15	15	1	15	15	1
5	D18S109	Chr18	29.81	15	15	15	15	1	15	15	1
6	D18S116	Chr18	43.04	17	17	17	17	1	17	17	1
7	D18S124	Chr18	44.76	29.2	29.2	29.2	29.2	1	29.2	29.2	1
8	D18S126	Chr18	62.05	16	16	16	16	1	16	16	1
9	D18S138	Chr18	67.63	20	20	20	20	1	20	20	1
10	D18S103	Chr18	69.07	15	15	15	15	1	15	15	1
11	D18S102	Chr18	83.51	39	39	39	39	1	39	39	1
1	D19S201	Chr19	8.76	16	16	16	16	1	16	16	1
2	D19S205	Chr19	16.13	17	17	17	17	1	17	17	1
3	D19S210	Chr19	33.04	21	21	21	21	1	21	21	1
4	D19S238	Chr19	49.53	20.3	20.3	20.3	20.3	1	20.3	20.3	1
5	D19S238	Chr19	53.57	20.3	20.3	20.3	20.3	1	20.3	20.3	1
1	DXS201	X	7.67	21.2		21.2		1	21.2		1
2	DXS207	X	18.46	18		18		1	18		1
3	DXS1107	X	40.66	23		23		1	23		1
4	DXS1106	X	46.64	38		38		1	38		1
5	DXS1301	X	59.07	18		18		1	18		1
6	DXS1319	X	73.53	23		23		1	22		0
7	DXS1304	X	83.42	19.2		19.2		1	19.2		1
8	DXS1308	X	96.71	15.3		15.3		1	15.3		1
9	DXS1312	X	104.29	18		18		1	18		1
10	DXS1405	X	117.87	28		28		1	28		1
11	DXS1602	X	134.74	19		19		1	19		1
12	DXS1608	X	138.09	22		22		1	22		1
13	DXS1713	X	150.95	17		17		1	17		1
14	DXS1705	X	159.41	16		16		1	16		1
15	DXS1709	X	166.49	19		19		1	19		1
1	DY101	Y	0.19	21		21		1	21		1
2	DY101I	Y	0.2	24		24		1	24		1

STR Marker Data				Analytical Results		Reference 1		Percent	Reference 2		Percent
				Probe 1 m		Jackson J		87.38%	Janvier Rj		86.92%
	Marker	Chr	Pos (Mbp)	S1, A1	S1, A2	R1, A1	R1, A2	S1-R1	R2, A1	R2, A2	S1-R2
3	DYS102	Y	0.33	24		24		1	24		1
4	DYS201	Y	0.94	28		28		1	28		1
5	DYS202	Y	1.73	14		14		1	14		1
6	DYS204	Y	2.57	20		20		1	20		1
7	DYS301	Y	3.99	16		16		1	16		1
8	DYS465	Y	46.6	15		15		1	15		1
9	DYS465I	Y	59.6	16		16		1	16		1
10	DYS601	Y	12.87	14		14		1	14		1
11	DYS601I	Y	17.38	16		16		1	16		1
12	DYS704	Y	17.38	18		18		1	18		1
14	DYS1001I	Y	18.38	7		7		1	7		1
13	DYS1001	Y	41.07	12		12		1	12		1
15	DYS1501	Y	81.16	11		11		1	11		1
16	DYS1501I	Y	35.69	12		12		1	12		1
17	DYS1501II	Y	36	17		17		1	17		1
