# Supplementary material for: Comparative phenotyping of C57BL/6J substrains reveals distinctive patterns of cardiac aging
Source: GeroScience. 2025 Jan 30;47(3):4795–812. doi: 10.1007/s11357-025-01543-7 (PMC12181499; doi:10.1007/s11357-025-01543-7)
Supplement: Supplementary file 4 — Supplementary file4 (PDF 291 KB) [file 11357_2025_1543_MOESM4_ESM.pdf]

Background check - BL/6 specific mutations

Sample receipt: 19.09.2024
Report Date: 24.11.2024

B6JCrl mice

1 m	Chromosom	Pos in MBp	Allel 1	Allel 2	Interpretation
**Rd8**	Chr 1	139,2	Mut	WT	Mutation typical for BL/6N
**Snca**	Chr 6	86,4	Mut	Mut	Mutation typical for BL/6JOlaHsd
**Dock 2**	Chr 11	34,2	WT	WT	Mutation typical for C57BL/6NHsd
**Nnt**	Chr 13	119,3	Mut	Mut	Mutation typical for BL/6J, BL/6JRj
**DIP686**	Chr 6	86,4	Mut	Mut	Mutation typical for BL/6J
**DIP1606**	Chr 16	6,1	Mut1	Mut1	Mutation 1 typical for BL/6
**Y Haplotype**	Chr Y		BL/6		BL/6

2 m	Chromosom	Pos in MBp	Allel 1	Allel 2	Interpretation
**Rd8**	Chr 1	139,2	Mut	WT	Mutation typical for BL/6N
**Snca**	Chr 6	86,4	WT	WT	Mutation typical for BL/6JOlaHsd
**Dock 2**	Chr 11	34,2	WT	WT	Mut typical for C57BL/6NHsd
**Nnt**	Chr 13	119,3	Mut	Mut	Mutation typical for BL/6J, BL/6JRj
**DIP686**	Chr 6	86,4	Mut	Mut	Mutation typical for BL/6J
**DIP1606**	Chr 16	6,1	Mut1	Mut1	Mutation 1 typical for BL/6
**Y Haplotype**	Chr Y		BL/6		BL/6

3 f	Chromosom	Pos in MBp	Allel 1	Allel 2	Interpretation
**Rd8**	Chr 1	139,2	WT	WT	Mutation typical for BL/6N
**Snca**	Chr 6	86,4	Mut	Mut	Mutation typical for BL/6JOlaHsd
**Dock 2**	Chr 11	34,2	WT	WT	Mutation typical for C57BL/6NHsd
**Nnt**	Chr 13	119,3	Mut	Mut	Mutation typical for BL/6J, BL/6JRj
**DIP686**	Chr 6	86,4	Mut	Mut	Mutation typical for BL/6J
**DIP1606**	Chr 16	6,1	Mut1	Mut1	Mutation 1 typical for BL/6
**Y Haplotype**	None				

B6JRj mice

4 m	Chromosom	Pos in MBp	Allel 1	Allel 2	Interpretation
**Rd8**	Chr 1	139,2	Mut	WT	Mutation typical for BL/6N
**Snca**	Chr 6	86,4	WT	WT	Mutation typical for BL/6JOlaHsd
**Dock 2**	Chr 11	34,2	WT	WT	Mutation typical for C57BL/6NHsd
**Nnt**	Chr 13	119,3	Mut	Mut	Mutation typical for BL/6J, BL/6JRj
**DIP686**	Chr 6	86,4	Mut	Mut	Mutation typical for BL/6J
**DIP1606**	Chr 16	6,1	Mut1	Mut1	Mutation 1 typical for BL/6
**Y Haplotype**	Chr Y		BL/6		BL/6

5 f	Chromosom	Pos in MBp	Allel 1	Allel 2	Interpretation
**Rd8**	Chr 1	139,2	WT	WT	Mutation typical for BL/6N
**Snca**	Chr 6	86,4	WT	WT	Mutation typical for BL/6JOlaHsd
**Dock 2**	Chr 11	34,2	WT	WT	Mutation typical for BL/6J, BL/6JRj
**Nnt**	Chr 13	119,3	Mut	Mut	Mutation typical for BL/6J/BL/6JRj
**DIP686**	Chr 6	86,4	Mut	Mut	Mutation typical for BL/6J
**DIP1606**	Chr 16	6,1	Mut1	Mut1	Mutation 1 typical for BL/6
**Y Haplotype**	None				

6 m	Chromosom	Pos in MBp	Allel 1	Allel 2	Interpretation
**Rd8**	Chr 1	139,2	Mut	WT	Mutation typical for BL/6N
**Snca**	Chr 6	86,4	WT	WT	Mutation typical for BL/6JOlaHsd
**Dock 2**	Chr 11	34,2	WT	WT	Mutation typical for C57BL/6NHsd
**Nnt**	Chr 13	119,3	Mut	Mut	Mutation typical for BL/6J/BL/6JRj
**DIP686**	Chr 6	86,4	Mut	Mut	Mutation typical for BL/6J
**DIP1606**	Chr 16	6,1	Mut1	Mut1	Mutation 1 typical for BL/6
**Y Haplotype**	Chr Y		BL/6		BL/6
